# Dietary and Serum Omega-6/Omega-3 Fatty Acids Are Associated with Physical and Metabolic Function in Stroke Survivors

**DOI:** 10.3390/nu12030701

**Published:** 2020-03-06

**Authors:** Monica C. Serra, Alice S. Ryan, Charlene E. Hafer-Macko, Manuel Yepes, Fadi B. Nahab, Thomas R. Ziegler

**Affiliations:** 1 San Antonio GRECC, South Texas Veterans Health Care System and Division of Geriatrics, Gerontology & Palliative Medicine and the Sam & Ann Barshop Institute for Longevity & Aging Studies, UT Health San Antonio, San Antonio, TX 78229, USA; 2 VA Maryland Health Care System and University of Maryland School of Medicine, Baltimore, MD 21201, USA; aryan@som.umaryland.edu (A.S.R.); chafermacko@som.umaryland.edu (C.E.H.-M.); 3 Emory University School of Medicine, Atlanta, GA 30322, USA; myepes@emory.edu (M.Y.); fnahab@emory.edu (F.B.N.); tzieg01@emory.edu (T.R.Z.)

**Keywords:** stroke, omega-3, omega-6, physical function, insulin resistance

## Abstract

The purpose of this study was to quantify habitual dietary and systemic omega-6 and omega-3 fatty acids and their ratios and to determine their relationship with physical and metabolic function in a cohort of chronic adult stroke survivors. Twenty-five older chronic stroke survivors (age: 63 ± 8 years; BMI: 31 ± 7 kg/m^2^; mean ± SD) were assessed for fitness (VO_2_peak), gait speed (GS), 3 m timed up and go (TUG), and six-minute walk distance (6MWD). Plasma lipid and glucose profiles were measured, and HOMA-IR calculated. Dietary (5-day food records) and serum (mass spectrometry) omega-6/omega-3 profiles were assessed. Participants were severely deconditioned (VO_2_peak: 19 ± 4 mL/kg/min; GS: 0.88 ± 0.28 m/s; TUG: 12.6 ± 5.9 s; 6MWD: 295 ± 121 m) and at elevated metabolic risk (HOMA-IR: 6.3 ± 4.5). The dietary intake ratio of omega-6/omega-3 fatty acids averaged 12.6 ± 7.1 and the serum concentration ratio was 1.21 ± 0.37, which were correlated (r = 0.88, *p* < 0.01). Higher dietary intake and serum concentrations of omega-6/omega-3 fatty acids were associated with lower 6MWD and higher HOMA-IR, while a higher serum omega-6/omega-3 concentration index was associated with lower VO_2_peak (*p*’s < 0.05). These preliminary data suggest that both dietary omega-6 and omega-3 fatty acids (quantitated as their intake ratio) and the serum concentration ratio of omega-6/omega-3 may be important indices of physical dysfunction and insulin resistance in chronic stroke survivors.

## 1. Introduction

Each year in the United States, ~800,000 people experience a stroke [1]. Poststroke declines in metabolic control (i.e., development of proinflammatory conditions, including hyperlipidemia, obesity, and insulin resistance) are common and contribute to strokes being a leading cause of long-term disability. Although sedentary activity is often implicated in these declines, less attention has been given to the role of dietary factors.

Current guidelines suggest that the dietary intakes of omega-3 and omega-6 long-chain polyunsaturated fatty acids (LC-PUFA) are important substitutes for saturated fatty acids, since their intake is associated with beneficial effects on cardiovascular risk [2]. Studies to date implicate higher dietary and systemic omega-6/omega-3 ratios in association with reduced physical functioning and elevated proinflammatory states in older adults [3] and neurological deterioration following acute stroke [4]. Despite being at elevated risk, the consumption and metabolic status of omega-6 and omega-3 fatty acids by chronic stroke survivors is not well documented. Though an optimal ratio has yet to be defined, the identification of omega-6/omega-3 as a clinical biomarker may have important implications for stroke recovery. Therefore, the purpose of this study was to examine the relationship between dietary and systemic omega-6/omega-3 and physical function and cardiovascular and diabetes risk in a cohort of chronic adult stroke survivors.

## 2. Materials and Methods

### 2.1. Population

This cross-sectional study included 25 chronic (>6 months) ischemic stroke survivors with hemiparetic gait between the ages of 45–80 years that were recruited from the Atlanta area. All volunteers signed Emory University IRB approved informed consents. Participants underwent a medical examination, which included height, weight, and resting blood pressure.

### 2.2. Physical Functioning

Exercise testing with open-circuit spirometry was conducted to measure cardiorespiratory fitness (VO_2_peak) using a graded treadmill test [5]. A standardized six-minute walk distance (6MWD) test recorded the distance walked at a comfortable self-selected pace in six minutes using the participant’s usual assistive device. The dynamic gait index assessment (DGI) was conducted to evaluate each participant’s ability to modify balance while walking in the presence of external demands (score of 0 to 24, with a higher score indicating less impairment) [6]. Gait speed was assessed from a 10 m walk conducted at usual walking speed. A 3 m timed up and go (TUG) was completed by having the participant walk to a line that is 3 m (9.8 feet) away, turn around at the line, walk back to the chair, and sit down as quickly as possible. Paretic and nonparetic handgrip strength were assessed using a portable handheld dynamometer. Fatigue was measured by the fatigue severity scale (score of 1 to 7, with a higher score indicating greater fatigue severity) [7].

### 2.3. Fatty Acid Dietary Intake

Average dietary and supplement caloric, macronutrient, and fatty acid intakes were calculated from five-day food records (three weekdays and two weekend days). Records were reviewed and analyzed by a Registered Dietitian using Nutrition Data System for Research software (Minneapolis, MN). Omega-3s were classified as α-linolenic (ALA-18:3), stearidonic (SDA-18:4), eicosapentaenoic (EPA-20:5), docosapentaenoic (DPA-22:5) and docosahexaenoic (DHA-22:6) acids, while omega-6s were classified as linoleic (LA-18:2) and arachidonic (AA-20:4) acids.

### 2.4. Blood Analyses

#### 2.4.1. Metabolic Functioning

Blood was drawn after a 12-h fast. Plasma glucose, lipid, and high sensitivity-C-reactive protein (hs-CRP) concentrations were determined by the colorimetric method and insulin by the immunoturbidometric method. Homeostatic Model Assessment of Insulin Resistance (HOMA-IR) was calculated as [(insulin (µU/mL) × glucose (mmol/L))/22.5] [8].

#### 2.4.2. Serum Fatty Acid Profiles

Lipids were extracted from 100 µL serum samples using a modified (100 µL 1M NaCl was used in place of water) Bligh and Dyer lipid extraction protocol [9]. Targeted lipidomics assays were conducted on a QTRAP 5500 LC-MS/MS system (ABSciex, Framingham, MA, USA) to quantitate selective omega-3 (EPA and DHA) and omega-6 (AA) lipid species by precursor ion scanning for the m/z values corresponding to the respective molecular weights. Calibration curves with standards showed a very good correlation between concentration and response. All calibration curves were reproducible.

#### 2.4.3. Statistical Analyses

Descriptive statistics were analyzed using SPSS (PASW Statistics, Version 24, Chicago, IL, USA). Results are expressed as mean ± SD with significance set at *p* < 0.05. Spearman correlation coefficients were used to determine relationships between dietary and serum omega-6/omega-3 and physical and metabolic outcomes.

## 3. Results

### 3.1. Physical and Metabolic Functioning

Participant characteristics and physical and metabolic functioning data are shown in Table 1. Subjects were 68% male and 56% African American. On average, subjects were obese, ~60 years old, and ~10 years poststroke. Participants, on average, had mild fatigue and poor physical functioning and weakness by VO_2_peak, DGI, gait speed, TUG, handgrip strength, and 6MWD. Treatment for hypertension and dyslipidemia occurred in 89% and 70%, respectively. Despite 44% being treated for diabetes, all participants had elevated fasting plasma glucose (≥100 mg/dL) and 88% had elevated HOMA-IR (≥2.5). Elevated hs-CRP (>3 mg/L) was observed in 24%.

### 3.2. Dietary and Serum Fatty Acid Profiles

No participant was taking supplements that contained omega-3 or omega-6 LC-PUFAs. The individual dietary and serum omega-3 and omega-6 fatty acid profiles are shown in Table 2. No differences between groups were noted for dietary or serum fatty acid profiles when evaluated by sex (male vs. female), ethnicity (African American vs. Caucasian), or age (<65 vs. ≥65 years) (data not shown). On average, stroke survivors consumed 8.9 ± 4.0 g/d of dietary omega-6s and 0.89 ± 0.54 g/d of dietary omega-3s, with an omega-6/omega-3 dietary intake ratio of 13 ± 7. Average serum omega-6 concentrations were 177 ± 38 g/d and serum omega-3 (EPA + DHA) concentrations were 153 ± 35 g/d, with an omega-6 (AA) to omega-3 (EPA + DHA) serum concentration ratio of 1.2 ± 0.4. Intakes below gender- and age-specific recommendations [2] occurred in 92% of participants for ALA and 52% of participants for LA. There was a significant association between dietary and serum omega-6/omega-3 ratios (r = 0.88, *p* < 0.01).

### 3.3. Associations between Dietary and Serum Fatty Acid Profiles and Functional and Cardiometabolic Risk

Associations between dietary and serum omega-6/omega-3 and functional and diabetes indices are shown in Table 3. Higher dietary and serum omega-6/omega-3 were associated with lower 6MWD, DGI, and handgrip strength, higher HOMA-IR, and increased serum hs-CRP concentration (*p*’s < 0.05). Additionally, a higher dietary omega-6/omega-3 was associated with a trend for a slower TUG and higher fasting glucose and insulin concentrations, while higher serum omega-6/omega-3 were associated with lower VO_2_peak (*p*’s < 0.05). No significant associations were found between dietary or serum omega-6/omega-3 and blood pressure (r’s = 0.16–0.26) or systemic cholesterol or triglyceride concentrations (r = 0.01–0.22).

## 4. Discussion

Though an optional serum omega-6/omega-3 has yet to be defined, a dietary intake ratio of (4–5)/1 is associated with a 70% decrease in total mortality and lower inflammation [10]. We find that, on average, this ratio is approximately three times higher in our sample of stroke survivors, which is twice as high as that observed in other older, but generally healthy, adult populations [11]. Further, this study provides novel preliminary evidence that greater dietary and serum omega-6/omega-3, signifying excess omega-6 LC-PUFAs intake and blood values relative to omega-3s, may adversely influence physical and metabolic health in chronic stroke survivors. While we are the first to examine these relationships in a cohort of chronic stroke survivors, this study supports data from non-stroke populations suggesting that higher dietary and systemic concentrations of omega-3 LC-PUFAs, compared to omega-6s, protect against accelerated age- and disease-associated decline of physical and psychosocial functioning and development of insulin resistance and chronic inflammation [12,13,14].

Despite studies identifying an association between acute poststroke plasma fatty acid profiles and stroke recurrence, dietary omega-3 supplementation trials in stroke survivors have been unsuccessful at reducing cardiovascular risk [15]. However, in stroke survivors receiving 250 mg/d of DHA and 250 mg/d of EPA orally for one year, a trend in greater improvements in functional status (i.e., activities of daily living and mobility disability) was observed [16]. Further, associations between reduction in serum omega-6/omega-3 and cholesterol-lowering and improvements in insulin sensitivity are observed in patients with cardiovascular disease following statin treatment [17]. These data suggest the need for further evaluation of dietary and systemic LC-PUFA modification on clinical outcomes beyond secondary prevention.

Consumption of omega-3 LC-PUFAs in the diet partially replaces the omega-6s in the membranes of most cells, including platelets, erythrocytes, neutrophils, monocytes, and hepatic cells [18]. Although our data are limited in this regard, a higher omega-3 membrane concentration may explain the associations between higher omega-6/omega-3 LC-PUFA profiles and physical and metabolic dysfunction in stroke survivors by altering cell membrane fluidity, enhancing nitric-oxide-mediated vasodilation, and attenuating platelet aggregation [19]. Further, there is some suggestion that the neuroprotective mechanism of action of omega-3s may occur through their effects on inflammation and oxidative stress; omega-6s produce eicosanoid products, which are more potent mediators of thrombosis and inflammation than similar products derived from omega-3s [20]. Although the exact mechanisms are unclear, these findings suggest that dietary and serum omega-3s might be important indicators to help attenuate and monitor the progression of stroke disability.

Although a larger study is needed to confirm our results, the use of both dietary and systemic LC-PUFA assessments in this pilot study strengthens the comprehensive evaluation of the relationships between habitual dietary omega-6 and omega-3 intake and their serum concentrations. Further, recent studies in stroke survivors identified systemic EPA and DHA concentrations similar to the current assessment, which were lower than in non-stroke controls [21,22], suggesting that these data may be generalizable to the larger U.S. stroke survivor population. However, the cross-sectional nature of the current study, with small sample size, limits the interpretation of the potential importance of covariates, including the latency of stroke and other comorbidities.

## 5. Conclusions

In summary, this preliminary report suggests that omega-6 and omega-3, measured via habitual dietary intake and serum concentrations, may be important indices of physical and metabolic dysfunction in chronic stroke survivors. Larger controlled trials are needed to investigate the optimal ratio of LC-PUFAs and the impact of their dietary modification to promote poststroke physical and metabolic recovery.

## Figures and Tables

**Table 1:** Participant characteristics.

*n* = 25	Mean ± SD	Ranges
M/F (*n*)	17/8	-
AA/C (*n*)	14/11	-
Age (years)	63 ± 8	45–82
BMI (kg/m^2^)	31 ± 7	19–45
Stroke Latency (months)	120 ± 52	8–144
VO_2_peak (mL/kg/min)	19 ± 4	12–25
6MWD (m)	295 ± 121	52–484
Dynamic Gait Index	17 ± 5	6–24
Usual Gait Speed (m/s)	0.88 ± 0.28	0.3–1.4
3 Meter Up and Go (s)	14.6 ± 5.9	7.6–31.8
Paretic Hand Grip Strength (kg)	26 ± 11	2–47
Nonparetic Hand Grip Strength (kg)	33 ± 11	14–61
Fatigue Severity Scale	3.7 ± 1.4	1.7–7
Systolic BP (mmHg)	128 ± 18	93–158
Diastolic BP (mmHg)	76 ± 10	57–99
Plasma Profiles		
Glucose (mg/dL)	144 ± 21	104–192
Insulin (µU/mL)	18 ± 13	6–57
HOMA-IR	6.3 ± 4.5	1.9–21.3
Total Cholesterol (mg/dL)	147 ± 55	72–334
Triglycerides (mg/dL)	63 ± 29	19–123
LDL Cholesterol (mg/dL)	92 ± 48	42–256
HDL Cholesterol (mg/dL)	46 ± 16	26–88
hs-CRP (mg/L)	4.2 ± 13.4	1.8–4.7

M: male; F: female; AA: African American; C: Caucasian; BMI: body mass index: 6MWD: 6 minute walk distance; BP: blood pressure; HOMA-IR: homeostatic model assessment of insulin resistance; LDL: low density lipoprotein; hs-CRP: high sensitivity-C-reactive protein.

**Table 2:** Dietary intake and serum fatty acid profiles.

*n* = 25	Mean ± SD	Ranges
Dietary Intake		
Energy (kcal/d)	1876 ± 821	1149–4274
Protein (g/d)	91 ± 45	50–257
Fat (g/d)	79 ± 40	33–179
Carbohydrate (g/d)	201 ± 89	105–451
Fatty Acid Profiles		
α-Linolenic Acid (g/d)	0.8 ± 0.5	0.08–1.95
Linoleic Acid (g/d)	8.8 ± 3.9	2.0–15.4
Arachidonic Acid (g/d)	109 ± 79	0–246
Stearidonic Acid (mg/d)	14 ± 7	0–27
Eicosapentaenoic Acid (mg/d)	24 ± 47	0–185
Docosahexaenoic Acid (mg/d)	71 ± 82	0–380
Docosapentaenoic Acid (mg/d)	13 ± 14	0–54
Serum Profiles		
Eicosapentaenoic Acid (μg/dL)	54 ± 24	20–112
Docosahexaenoic Acid (μg/dL)	99 ± 21	68–140
Arachidonic Acid (μg/dL)	177 ± 38	117–279

**Table 3:** Relationships between omega-6/omega-3 dietary intake and serum concentrations and physical function and diabetes risk.

Pearson Coefficients	Dietary Intake	Serum
VO_2_peak (mL/kg/min)	−0.25	−0.38 *
6MWD (m)	−0.49 **	−0.43 *
Dynamic Gait Index	−0.86 **	−0.70 **
Usual Gait Speed (m/s)	0.39	0.27
3 Meter Up and Go (s)	0.41 *	0.35
Paretic Hand Grip Strength (kg)	−0.61 **	−0.64 **
Nonparetic Hand Grip Strength (kg)	−0.64 **	−0.59 **
Fatigue Severity Scale	0.18	0.12
Glucose (mg/dL)	0.37 *	0.18
Insulin (µU/mL)	0.34 *	0.34
HOMA-IR	0.39 *	0.34 *
hs-CRP (mg/L)	0.42 *	0.48 **

*p* = 0.06; * *p* < 0.05; ** *p* < 0.01.
